# Study on the Microstructure Evolution and Ablation Mechanism of SiC_p_/Al Composites Processed by a Water-Jet Guided Laser

**DOI:** 10.3390/ma18122749

**Published:** 2025-06-11

**Authors:** Wendian Yin, Ze Yu, Guanghao Xing, Feng Yang, Zhigang Dong

**Affiliations:** 1 Chengdu Aircraft Industry (Group) Co., Ltd., Chengdu 610000, China; 2 State Key Laboratory of High-Performance Precision Manufacturing, Dalian University of Technology, Dalian 116024, China

**Keywords:** SiC_p_/Al composites, water-jet guided laser, microgroove, removal mechanism

## Abstract

In this study, the influence of different process parameters on the macroscopic and microscopic morphology of the microgroove in the water-jet guided laser was studied. In addition, the microstructure evolution and material ablation mechanism of the microgroove were studied. The results show that with the increase in laser power, the depth of the microgroove increases from 154 μm to 492 μm, the width from 63 μm to 74 μm, and the depth-to-width ratio from 2.45 to 6.62; with the increase in scanning speed, the depth of the microgroove decreases from 525.33 μm to 227.16 μm, and the width from 67.61 μm to 71.02 μm, and the depth-to-width ratio from 7.77 to 3.20. With the increase in water jet pressure, the depth increases from 312.29 μm to 3.20. With the increase in water jet pressure, the depth increased from 312.29 μm to 362.39 μm, the width decreased from 71.59 μm to 62.78 μm, and the depth-to-width ratio increased from 4.38 to 5.77. In addition, the water guided laser processing of SiC_p_/Al composites produces thermal–mechanical coupling and chemical reaction synergies: the material melts and vaporizes under the action of a high-energy laser beam, and the SiC particles are oxidized and thermally decomposed at local high temperatures due to their high thermal stability.

## 1. Introduction

Silicon carbide particle-reinforced aluminum matrix (SiC_p_/Al) composites are widely used in aerospace, automotive, and electronics due to their outstanding material properties, including high specific strength, excellent specific stiffness, low thermal expansion coefficient, and superior wear resistance [1,2]. To meet assembly requirements, machining operations, such as contour cutting and hole drilling, are typically required for SiC_p_/Al composites blanks. Given the heavy loading conditions and harsh operating environments of advanced weapon systems, SiC_p_/Al components must achieve exceptionally high machining quality and precision to ensure reliable performance under extreme mechanical stresses [3,4,5].

However, the significant mismatch in mechanical and thermophysical properties between SiC particles and the Al matrix, combined with the abrasive nature of the particles and the high ductility of the aluminum, makes SiC_p_/Al composites extremely difficult to machine, classifying them as a typical hard-to-cut material [2,6]. In light of this, researchers worldwide have conducted extensive studies on machining SiC_p_/Al composites. Currently, conventional contact-based techniques, such as milling [7], turning [8], grinding [9], and ultrasonic-assisted machining [10], have been conducted.

The aforementioned studies have contributed to advancing machining technologies for SiC_p_/Al composites, yet several critical issues remain unresolved. First, under tool interaction, machined surfaces suffer from particle-related damage, such as fragmentation, pull-out, and interfacial debonding, along with matrix defects including surface cracks, pits, and smearing, all of which degrade machining quality. Second, the abrasive nature of SiC particles and the high ductility of the Al matrix induce periodic fluctuations in cutting forces when using diamond tools, leading to rapid tool wear. Frequent tool replacements are required to maintain precision, significantly reducing machining efficiency and prolonging development cycles. Finally, thermally softened matrix material can mask surface pits and cracks, creating a false improvement in surface roughness. However, the weak bonding strength between smeared material and underlying defects means such concealed damage still compromises fatigue life and mechanical performance [11]. These latent defects critically undermine component reliability in service environments. Although theoretical modeling and process optimization have mitigated such damage to some extent, conventional contact-based machining techniques remain fundamentally incapable of fully eliminating these issues. Thus, there is an urgent need to develop novel non-contact machining methods to overcome these challenges.

Water-jetguided laser (WJGL) is a hybrid processing technology that combines a laser with water jet, where the water jet action significantly reduces the heat-affected zone, decreases residual stresses, and minimizes microcracks while its flushing effect effectively limits molten material accumulation, reduces recast layers, and debris deposition, thereby improving machining quality. Due to its advantages. Including deep processing capacity, small heat-affected zone, as well as high machining quality and precision, this technology has been widely applied in the contour cutting and hole drilling of difficult-to-machine materials, prompting extensive research by scholars worldwide. Tan researched the water-jet guided laser (WJGL) machining of SiC_p_/Al composites, performing comparative experiments between the conventional laser and WJGL in drilling and slotting, and analyzed the experimental results and machining quality. The results demonstrate that WJGL exhibits significant superiority. Yuan et al. [12,13] investigated WJGL machining of microgrooves and microholes in laminated SiC_f_/SiC composites, analyzing the post-machined micromorphology and material phases. Their findings indicate that the water jet effectively suppresses or even eliminates oxidation, achieving high-precision machining quality in microgrooves and microholes. Cheng et al. [14] conducted experimental studies on the WJGL cutting of SiC_f_/SiC composites, demonstrating that the cut sections exhibited no issues, such as a recast layer, SiC fiber pull-out, or delamination. SiC_p_/Al and SiC_f_/SiC composites have high processing challenges, highlighting the advanced capabilities of water-jet guided laser processing technology, and have different characteristics and application scenarios. SiC_p_/Al composites possess excellent properties such as low density, high specific strength and stiffness, and high elastic modulus, and have been widely applied in fields like aerospace, advanced weapon systems, electronic packaging, and automotive manufacturing. SiC_f_/SiC composites possess excellent oxidation resistance, ablation resistance, and wear resistance, and are widely used in hot-end components, such as turbine blades and combustion chamber flame tubes of aero engines.

In short, water-jet guided laser technology has significant advantages in difficult machining and material processing, but related research mainly focuses on C_f_/SiC and SiC_f_/SiC materials, and there are few related studies on SiC_p_/Al composites. In addition, the profile processing quality and accuracy of SiC_p_/Al composites components are crucial to assembly accuracy and service reliability, but there are no relevant reports on SiC_p_/Al composites processed by water-jet guided laser with different process parameters, and there is a lack of relevant research on processing technology and other depth-to-widths. Therefore, it is urgent to carry out research on the processing quality of SiC_p_/Al composites with different process parameters to improve service performance. Therefore, it is urgently necessary to carry out the influence laws of different process parameters on the processing quality of microgrooves of SiC_p_/Al composites and reveal the removal mechanism of SiCp/Al composites processed by a water-jet guided laser.

Based on the above, this paper designed an experiment to investigate the influence of different process parameters (laser power, scanning speed, water jet pressure) on the variation trends of microgroovedepth, width, and depth-to-width ratio. Additionally, the surface morphology evolution, ablation byproducts, and microstructural changes were analyzed using scanning electron microscopy (SEM), energy-dispersive spectroscopy (EDS), and Raman spectroscopy. Finally, the ablation mechanisms of the water-jetguided laser (WJGL) machining of SiC_p_/Al composites microgrooves were summarized, providing a theoretical foundation for subsequent process optimization.

## 2. Experimental Setup

### 2.1. SiC_p_/Al Composites

The material used in this experiment is SiC_p_/Al composites prepared by a pressure infiltration method. The matrix phase is 2024 aluminum alloy, and the reinforcing phase is abrasive grade SiC particles. The physical characteristics parameters of the SiC_p_/Al composites are shown in Table 1, which is provided by the material manufacturer.

**Figure 1:**
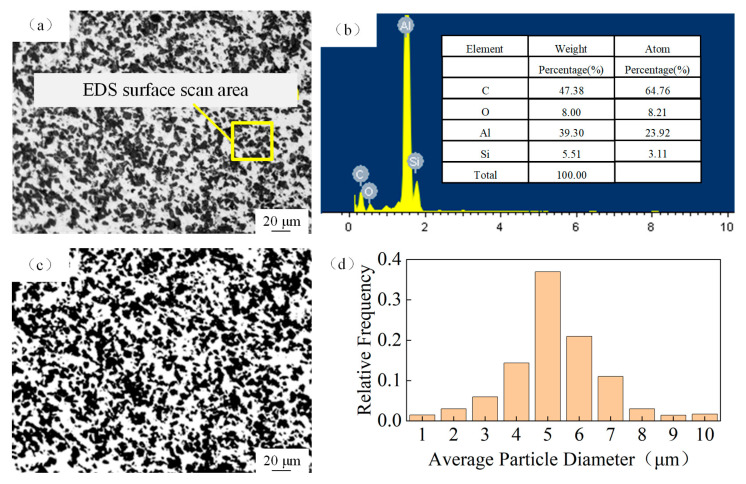
Microstructure of SiC_p_/Al composites. (**a**) Metallographic microstructure; (**b**) elemental content; (**c**) binary image; (**d**) distribution of average particle diameter.

In order to determine the particle size distribution, the SiC_p_/Al composite samples were first ground and polished. The metallographic microstructure of the material was observed using an optical microscope (LEICA DMi8) as shown in Figure 1a. The result of the elemental content conducted by EDS is presented in Figure 1b, for which the main elements are Si, Al, and C. Then, the image metallographic photographs were processed with the help of image processing technology and combined with statistical analysis to determine the particle size distribution pattern. The metallographic micrographs were processed with the help of image deblurring and binarization, and the results are shown in Figure 1c. By identifying and counting the number of pixels inside a single particle and the number of pixels on the outer contour of the particle, the area S_sic-1_ of a single particle and the perimeter P_sic-1_ of the particle was determined, and then the equivalent size of the particle was used to describe the size of irregular particles [15]; based on the law of large numbers in the theory of probability, and with the guarantee of an error range of 0.05 and a reliability of 90%, the number of samples of particles that need to be counted is z = 9000. The final determination of the average size of particles R, can be expressed as Equation (1). The result of the particle size distribution is shown in Figure 1d, in which the average particle size is 5.1 μm.

(1) $$R=\frac{\sum_{1}^{Z}\frac{4S_{sic-1}}{P_{sic-1}}}{2Z}$$

### 2.2. Equipment and Procedure

**Figure 2:**
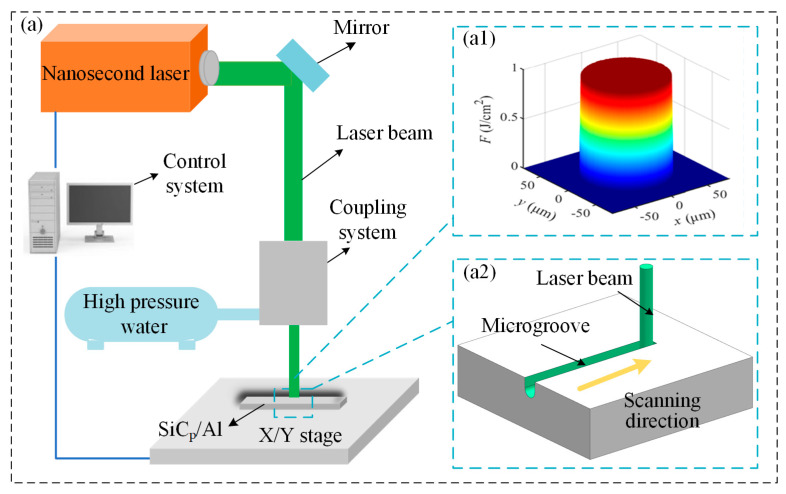
Water-jet guided laser processing equipment. (**a**) Experimental platform; (**a1**) flat-top beam distribution; (**a2**) scanning schematics.

The water-jetguided laser ablation experiments on SiC_p_/Al composites were conducted on the laser processing platform. The water-jetguided laser ablation experiment of SiC_p_/Al composites was carried out on the laser processing platform. As shown in Figure 2a, the water jetguided laser processing equipment is composed of a control system, a coupling system, a high-pressure water system, a workpiece worktable, etc. The water-jet guided laser coupling energy beam was a flat-topped beam (Figure 2a1), and the SiC_p_/Al composites were scanned using an X/Y moving stage (Figure 2a2). The nanosecond laser parameters and processing conditions used in the experiment are detailed in Table 2 and Table 3, respectively. The effects of average laser power, scanning speed, and water jet pressure on the quality of the microgroove were investigated. This approach aims to identify the preferred parameter range for process optimization. All scanning tests were conducted at room temperature in a protected environment without the presence of gas.

### 2.3. Characterization

After the microgroove treatment of SiC_p_/Al samples, the microgrooves were photographed and obtained using a confocal microscope (Olympus, OLS5100, Dalian, China), which has a lateral resolution of 0.12 μm and a height resolution of 6 nm. The instrument is able to quickly and efficiently complete the precise measurement of sub-micron morphology and surface roughness with an auto-calibration function, and in addition, the surface morphology of the microgrooves was examined using an electron microscope (HITACHI SU5000, Dalian, China).

## 3. Results and Discussion

### 3.1. Microgroove Quality

#### 3.1.1. Effects of Laser Power

**Figure 3:**
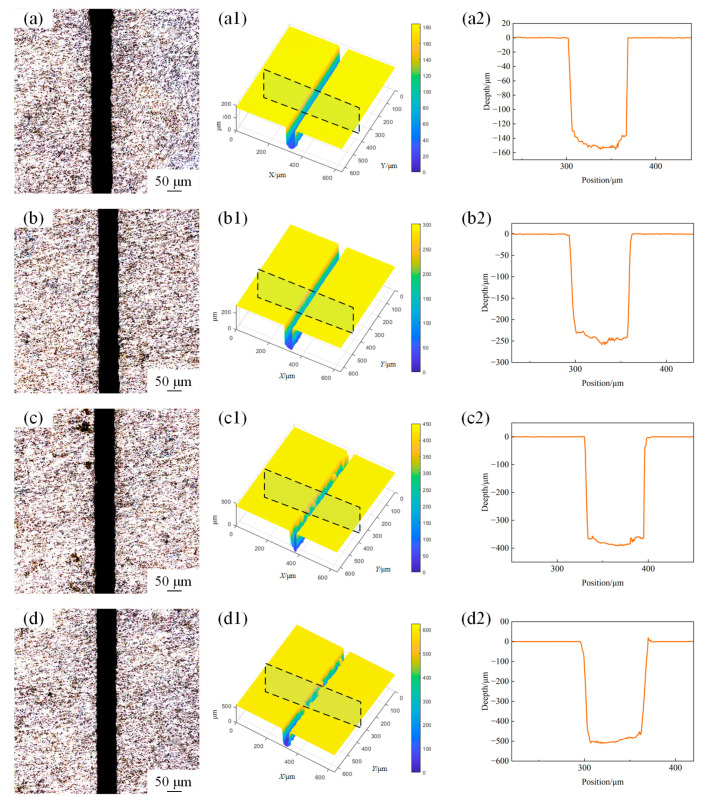
The surface morphology of microgroove under different powers. (**a**) 5 W; (**b**) 10 W; (**c**) 15 W; (**d**) 20 W. (**a1**) 3D profile of microgroove at 5 W; (**b1**) 3D profile of microgroove at 10 W, (**c1**) 3D profile of microgroove at 15 W; (**d1**) 3D profile of microgroove at 20 W; (**a2**) 2D cross-section profile of microgroove at 5 W; (**b2**) 2D cross-section profile of microgroove at 10 W; (**c2**) 2D cross-section profile of microgroove at 15 W; (**d2**) 2D cross-section profile of microgroove at 20 W.

**Figure 4:**
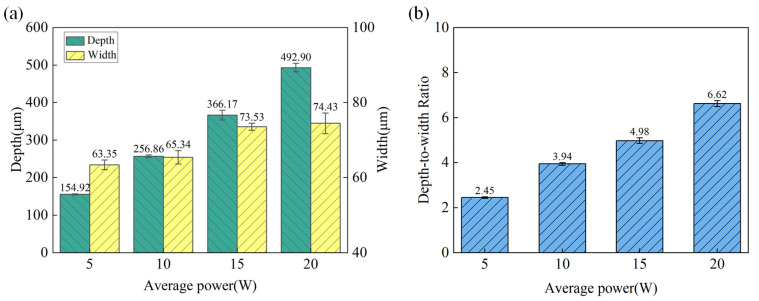
The variation in microgroove geometric characteristics under different laser powers. (**a**) Depth and width. (**b**) Depth-to-width ratio.

Microgroove morphology was obtained using confocal microscopy with a sampling area of 600 μm × 600 μm. The surface morphology of the microgroove under different laser powers with a scanning speed of 1mm/s and water jet pressure of 20 MPa is shown in Figure 3. Figure 4a illustrates the variation trends of the depth and width of the machined microgroove under different laser power settings. The depth and width of the microgroove exhibit significant trends during the machining process as the laser power varies. With the increase in laser power, the depth of the microgroove significantly increases. This is because a higher laser power delivers more energy to the material, thereby enhancing the melting and material removal effects. Simultaneously, the width of the microgroove also shows an increasing trend, though the rate of increase is relatively smaller. This is primarily due to the combined effects of the energy distribution of the laser beam and the thermal diffusion within the material [16]. As shown in Figure 4b, the depth-to-width ratio (depth-to-width ratio) exhibits an increasing trend with the rise in laser power. This is because the increase in depth is more pronounced compared to the increase in width, resulting in a higher depth-to-width ratio as the laser power intensifies. This behavior can be attributed to the focused energy distribution of the laser beam, which leads to deeper material penetration while maintaining a relatively controlled lateral expansion. At lower power levels, both the depth and width are relatively small, resulting in a lower depth-to-width ratio, and the increase in the depth-to-width ratio is relatively gradual. As the laser power further increases, the growth of the depth-to-width ratio becomes more pronounced, this is due to the higher energy density of the laser beam, which drives non-linear growth in the depth direction, enabling the material to penetrate more deeply. At the same time, due to limited lateral thermal diffusion and the top-hat energy distribution of the laser beam (which confines material removal to a more localized area), the width remains relatively stable. As a result, the depth-to-width ratio increases significantly.

#### 3.1.2. Effects of Scanning Speed

**Figure 5:**
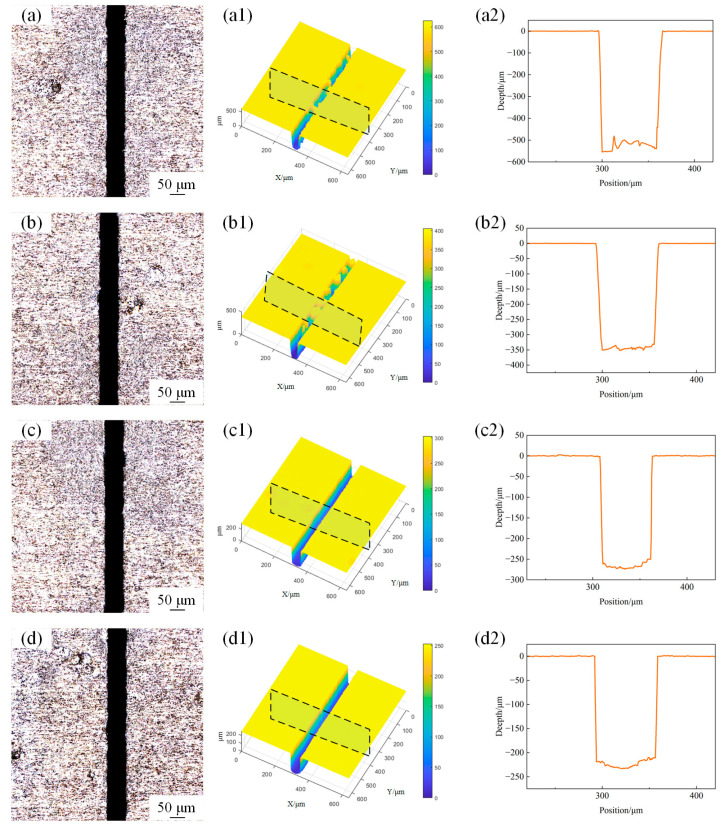
The surface morphology of the microgroove at different speeds. (**a**) 0.5 mm/s; (**b**) 1 mm/s; (**c**) 1.5 mm/s; (**d**) 2 mm/s; (**a1**) 3D profile of microgroove at 0.5 mm/s; (**b1**) 3D profile of microgroove at 1.0 mm/s, (**c1**) 3D profile of microgroove at 1.5 mm/s; (**d1**) 3D profile of microgroove at 2.0 mm/s; (**a2**) 2D cross-section profile of microgroove at 0.5 mm/s; (**b2**) 2D cross-section profile of microgroove at 1.0 mm/s; (**c2**) 2D cross-section profile of microgroove at 1.5 mm/s; (**d2**) 2D cross-section profile of microgroove at 2.0 mm/s.

**Figure 6:**
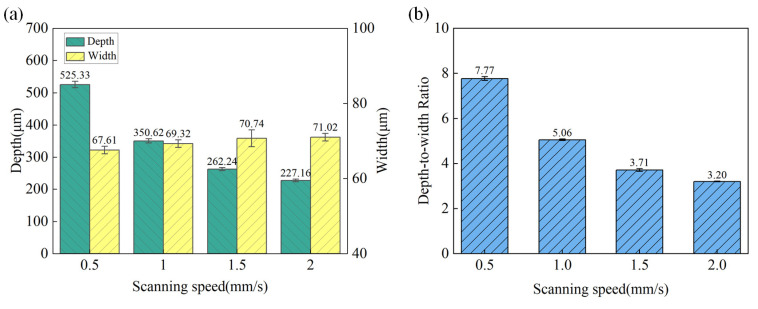
The variation in microgroove geometric characteristics under different scanning speeds. (**a**) Depth and width; (**b**) depth-to-width ratio.

The surface morphology of the microgroove under different scanning speeds with a laser power of 15 W and water jet pressure of 20 MPa is shown in Figure 5. The variation trends of the depth and width of the machined microgroove at different scanning speeds are shown in Figure 6a. As can be observed, with the increase in scanning speed, the depth of the microgroove significantly decreases. This is attributed to the higher scanning speed reducing the laser’s interaction time per unit area, resulting in decreased energy input and a reduction in the extent of material melting and vaporization. In addition, the increase in scanning speed narrows the lateral energy distribution range of the laser beam, resulting in a slight reduction in the width of the microgroove as the scanning speed increases. However, the magnitude of this change is more gradual compared to the decrease in depth. As shown in Figure 6b, since the depth decreases rapidly with the increase in scanning speed, while the change in width is relatively small, the depth-to-width ratio exhibits an overall declining trend. At low scanning speeds, the depth-to-width ratio is relatively high; however, as the scanning speed increases, the depth-to-width ratio gradually decreases. In the laser processing of microgrooves, it is essential to select the appropriate scanning speed based on specific requirements: low speeds are suitable for achieving high depth-to-width ratios and high precision, while high speeds are more applicable for rapid, batch processing with relatively lower precision demands. By optimizing the scanning speed, a balance can be struck between processing efficiency and microgroove quality, ensuring that the desired outcomes are achieved effectively.

#### 3.1.3. Effects of Water Jet Pressure

**Figure 7:**
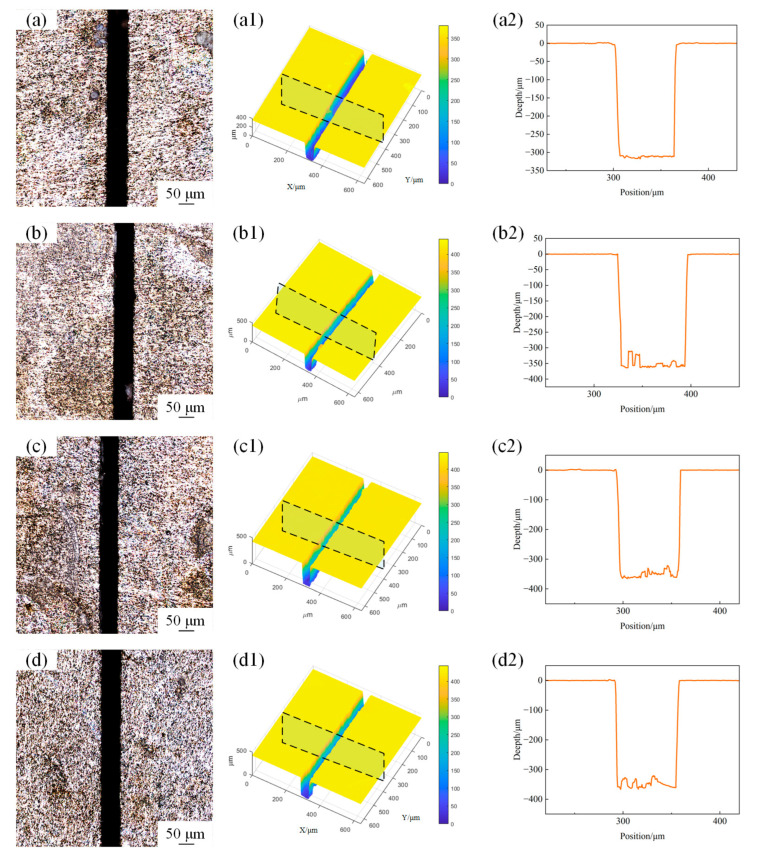
The surface morphology of microgroove at different water jet pressures. (**a**) 15 MPa; (**b**) 20 MPa; (**c**) 25 MPa; (**d**) 30 MPa; (**a1**) 3D profile of microgroove at 15 MPa; (**b1**) 3D profile of microgroove at 20 MPa, (**c1**) 3D profile of microgroove at 25 MPa; (**d1**) 3D profile of microgroove at 30 MPa; (**a2**) 2D cross-section profile of microgroove at 15 MPa; (**b2**) 2D cross-section profile of microgroove at 20 MPa; (**c2**) 2D cross-section profile of microgroove at 25 MPa; (**d2**) 2D cross-section profile of microgroove at 30 MPa.

**Figure 8:**
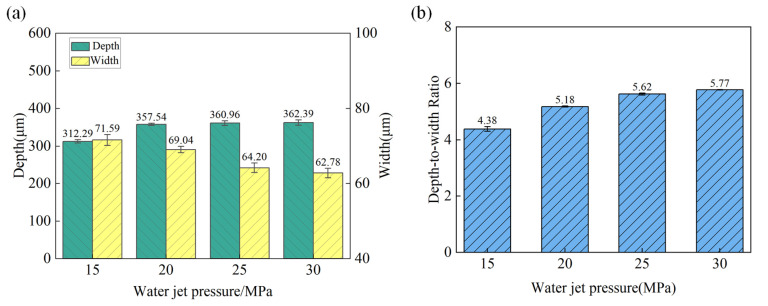
The variation in microgroove geometric characteristics under different water jet pressures. (**a**) Depth and width; (**b**) depth-to-width ratio.

The surface morphology of the microgroove under different water jet pressures with laser power 15 W and a scanning speed of 1 mm/s is shown in Figure 7. The variation trends of the depth and width of the machined microgroove at different water jet pressures are shown in Figure 8a. As the water jet pressure increases, the depth of the microgroove tends to grow, primarily driven by the enhanced kinetic energy of the water jet. On the contrary, the width tends to decrease. As the water pressure increases, the flow rate of the water jet per unit time rises, enhancing the cooling effect on the microgroove within the same timeframe. This effectively suppresses the spread of laser thermal influence in a timely manner. Figure 8b illustrates the variation in the depth-to-width ratio of the microgroove with the changes in water jet pressure. Within the range from low to moderate pressure, the depth-to-width ratio increases as the water jet pressure rises. However, under high-pressure conditions, due to the reduction in width, the depth-to-width ratio tends to stabilize, and the rate of increase diminishes.

### 3.2. Microstructure Evolution

**Figure 9:**
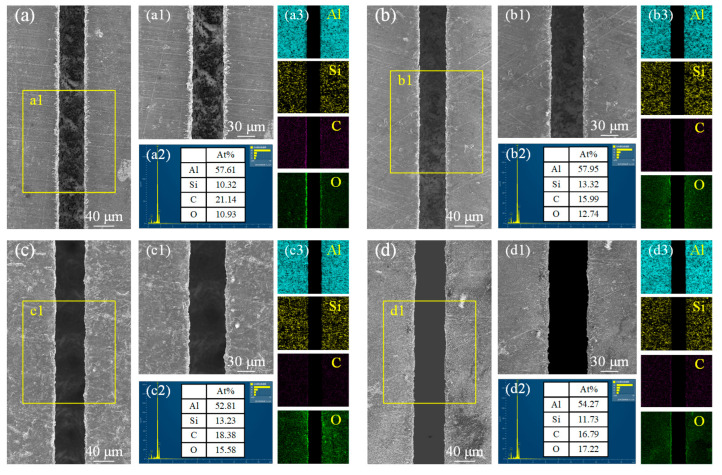
Microstructural morphology changes and EDS analysis of microgroove at different power Levels. (**a**) 5 W; (**b**) 10 W; (**c**) 15 W; (**d**) 20W; (**a1**) local enlargement of (**a**); (**b1**) local enlargement of (**b**); (**c1**) local enlargement of (**c**); (**d1**) local enlargement of (**d**); (**a2**) Elemental content of C, Si, O and Al at 5 W; (**b2**) Elemental content of C, Si, O and Al at 10 W; (**c2**) Elemental content of C, Si, O and Al at 15 W; (**d2**) Elemental content of C, Si, O and Al at 20 W; (**a3**) Elemental distribution of Al, Si, C, and O at 5 W; (**b3**) Elemental distribution of Al, Si, C, and O at 10 W; (**c3**) Elemental distribution of Al, Si, C, and O at 15 W; (**d3**) Elemental.

The high-resolution scanning electron microscope was used to further investigate the microstructure evolution of the ablated areas under different machining parameters. Figure 9 illustrates the evolution of the microstructure on the groove surface under different laser powers. As shown in Figure 9a, at a power of 5 W, the bottom of the microgroove can be clearly observed, while some re-solidified materials are generated on both sides of the microgroove. This occurs because during the processing, the laser ablates the SiC_p_/Al surface, and the material absorbs the laser energy, leading to melting and vaporization. Subsequently, under the impact and cooling effects of the water flow, these molten materials rapidly solidify and adhere to the sides of the microgroove. The EDS analysis results reveal that the oxygen atomic percentage in the ablation products is 10.93%, indicating that during the laser ablation process, the material reacts with the surrounding oxygen, resulting in the formation of oxides. As shown in Figure 9c,d, when the laser power is increased to 15 W and 20 W, the ablation effect of the material is further enhanced. The significant increase in energy density leads to the more efficient absorption of laser energy by the SiC_p_/Al surface, resulting in a substantial increase in the ablation rate. Consequently, the depth and width of the microgroove further expand. At 15 W and 20 W, the interaction between the laser and the material becomes more intense, and the amount of molten material generated increases significantly. However, due to the larger depth and width of the microgroove, the interaction area between the molten material and the water flow expands, causing some of the molten material to be washed away by the water flow. This results in a further reduction in the re-solidified material on both sides of the microgroove. Nevertheless, as the power increases, the oxidation reaction on the material surface becomes more pronounced. The EDS analysis reveals that the oxygen atomic percentages are 15.58% and 17.22%, respectively, which are higher than the oxygen atomic percentage at 10 W, indicating an intensified degree of oxidation. Additionally, the heat-affected zone (HAZ) expands at higher power levels.

**Figure 10:**
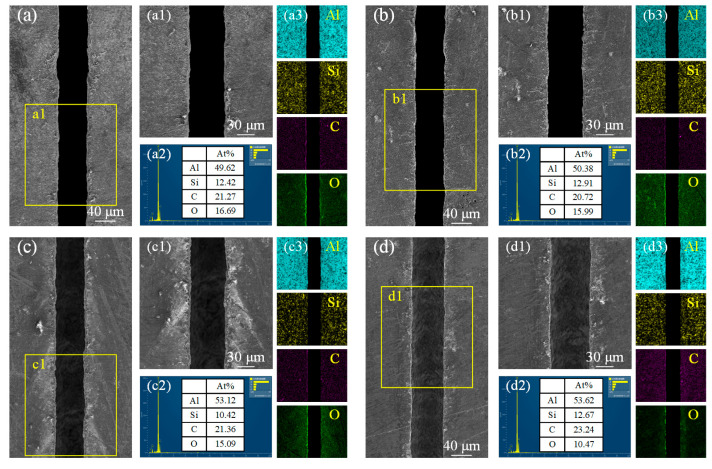
Microstructural morphology changes and EDS analysis of microgroove at different scanning speeds. (**a**) 0.5 mm/s; (**b**) 1 mm/s; (**c**) 1.5 mm/s; (**d**) 2 mm/s. (**a1**) local enlargement of (**a**); (**b1**) local enlargement of (**b**); (**c1**) local enlargement of (**c**); (**d1**) local enlargement of (**d**); (**a2**) Elemental content of C, Si, O and Al at 0.5 mm/sr; (**b2**) Elemental content of C, Si, O and Al at 1 mm/s; (**c2**) Elemental content of C, Si, O and Al at 1.5 mm/s; (**d2**) Elemental content of C, Si, O and Al at 2 mm/s; (**a3**) Elemental distribution of Al, Si, C and O at 0.5 mm/s; (**b3**) Elemental distribution of Al, Si, C and O at 1 mm/s; (**c3**) Elemental distribution of Al, Si, C and O at 1.5 mm/s; (**d3)** Elemental distribution of Al, Si, C and O at 2 mm/s.

The variations in microgroove morphology and elemental content at different scanning speeds are illustrated in Figure 10. When the scanning speeds are set to 0.5 mm/s and 1 mm/s (Figure 10a,b), the lower scanning velocity allows the material more time to absorb energy, resulting in a deeper microgroove. The processed microgroove exhibits smoother and more regular profiles on both sides, with clearly defined edges. As shown in Figure 10c,d, the increase in scanning speed leads to a shorter interaction time between the laser and the material surface. Rapid cooling results in the formation of finer grain structures at the edges of the microgroove, but it also increases the accumulation of thermal stress, leading to an irregular surface morphology of the microgroove. The increase in scanning speed reduces the stability of the microgroove melt pool, causing material spattering, irregular protrusions, and residual splatter at the edges of the microgroove. Combined with EDS analysis, it was found that as the scanning speed increases, the percentage of oxygen content gradually decreases. This is because a lower scanning speed results in a longer interaction time between the laser and the material, leading to higher temperatures in the affected region, which makes the material more prone to react with oxygen. As the speed increases, the interaction time becomes shorter, reducing the extent of the reaction between the material and oxygen.

**Figure 11:**
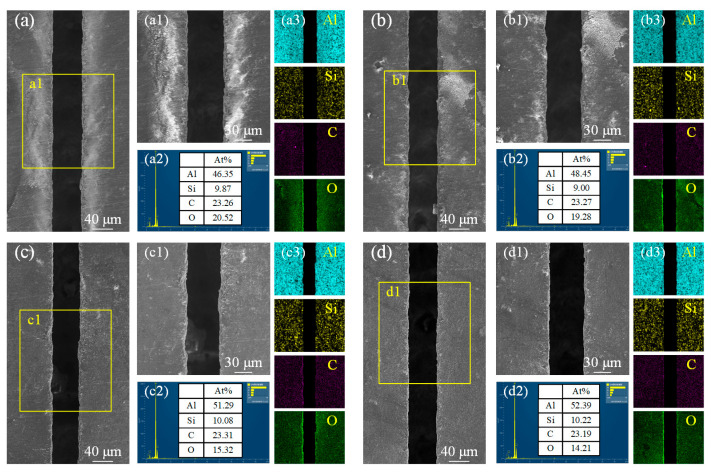
Microstructural morphology changes and EDS analysis of microgroove at different water jet pressures. (**a**) 15 MPa; (**b**) 20 MPa; (**c**) 25 MPa; (**d**) 30 MPa. (**a1**) local enlargement of (**a**); (**b1**) local enlargement of (**b**); (**c1**) local enlargement of (**c**); (**d1**) local enlargement of (**d**); (**a2**) Elemental content of C, Si, O and Al at 15 MPa; (**b2**) Elemental content of C, Si, O and Al at 20 MPa; (**c2**) Elemental content of C, Si, O and Al at 25 MPa; (**d2**) Elemental content of C, Si, O and Al at 30 MPa; (**a3**) Distribution of Al, Si, C, and O elements at 15 MPa; (**b3**) Distribution of Al, Si, C, and O elements at 20 MPa; (**c3**) Distribution of Al, Si, C, and O elements at 25 MPa; (**d3**) Distribution of Al, Si, C, and O elements at 30 MPa.

The morphology and elemental composition changes of the microgroove under different water jet pressures are shown in Figure 11. At a water jet pressure of 15 MPa, the relatively low pressure prevents the timely cooling and removal of material after laser processing. As a result, the surface morphology on both sides of the microgroove may appear relatively rough, with significant amounts of molten material residue and oxidation traces observed (Figure 11a). According to Figure 11a1), the oxygen content percentage reaches as high as 20.52%, indicating that oxidation reactions have occurred and formed oxide substances on both sides of the microgroove. As shown in Figure 11b, with the increase in water jet pressure, the energy transfer efficiency improves, resulting in a more uniform surface morphology on both sides of the microgroove. Although the oxide layer remains, the amount of residual molten material is reduced. According to Figure 11b1, the oxygen content percentage slightly decreases. This suggests that higher water jet pressure enhances material removal and reduces oxidation, leading to better surface quality. As shown in Figure 11c, when the water jet pressure reaches 25 MPa, the scouring effect of the water jet becomes significantly more pronounced. On both sides of the microgroove, the white oxide substances are nearly eliminated. At this point, the oxygen content percentage decreases to 15.32%, as seen in Figure 11c1. When the water jet pressure reaches 30 MPa (Figure 11d), the morphology on both sides of the microgroove becomes the smoothest and most uniform, with almost no residual molten material, as shown in Figure 11d1. The oxygen content percentage decreases further to 14.21%, indicating that the oxidation reaction is further suppressed. The oxides on both sides of the microgroove are nearly eliminated. Throughout the entire process, as the water jet pressure progressively increases, the scouring capability of the water jet on the molten material is enhanced. This effectively reduces the adherence of molten material to the sidewalls of the microgroove. Concurrently, the reduction in oxygen content prevents the formation of oxide layers, thereby further optimizing the surface morphology on both sides of the microgroove.

### 3.3. Ablative Mechanism

**Figure 12:**
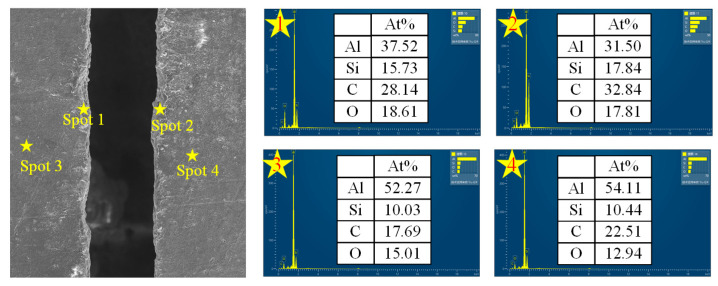
The microscopic morphology of the microgroove and the change trend of element content on both sides.

As shown in Figure 12, the deposited material on both sides of the microgroove exhibits a decrease in Al content and an increase in C, Si, and O content. When the water-guided laser interacts with the surface of the SiCp/Al composite material, the high-energy laser beam induces localized heating, leading to a rapid temperature rise. Due to the differences in the physical and chemical properties between SiC particles and the Al matrix, they undergo distinct chemical reactions under the influence of the laser. First, the Al matrix melts or even vaporizes under high temperatures (2). This causes some Al to evaporate as steam, while another portion reacts with oxygen to form Al_2_O_3_ (3). However, under the impact of the water jet, part of the oxide residue and other byproducts are removed, leading to a reduction in Al content. With further increasing temperature, silicon carbide (SiC) first reacts with oxygen to form solid SiO_2_ and CO_2_ (4). Due to the low volatility of SiO_2_ in its solid state, it tends to accumulate in the deposited material. When the temperature rises again, silicon carbide (SiC) partially decomposes, generating Si vapor and C vapor (5). The carbon (C) after the decomposition of silicon carbide exists in its elemental form, resulting in an increase in carbon content. The reason for this phenomenon is that under high-temperature conditions, silicon carbide (SiC) decomposes. The melting and boiling points of silicon (Si) are much lower than those of carbon (C), making it easier for Si atoms to escape from the surface [12].

Al(l)→Al(g)(2)

4Al(l) + 3O_2_→2Al_2_O_3_(s) (3)

SiC(s)+2O_2_→SiO_2_(s) + CO_2_ (4)

SiC(s)→Si(g) + C(g)(5)

**Figure 13:**
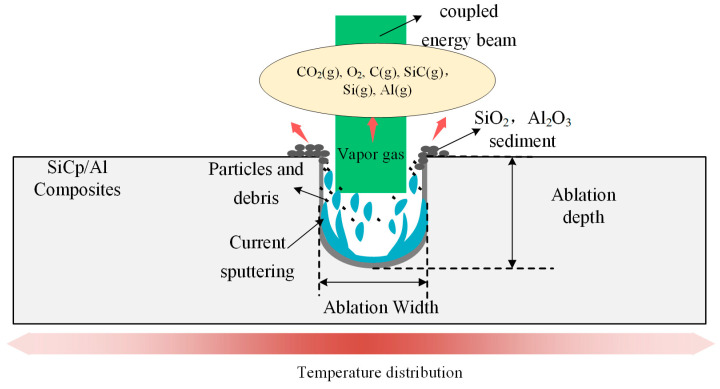
Ablation and removal mechanism of SiC_p_/Al composites under the action of a water-jet-guided laser.

As shown in Figure 13, Under the action of the water-jet guided laser, the removal mechanism of SiC_p_/Al composites is shown in the figure. The microgroove ablation mechanism of SiC_p_/Al composites processed by the water-jet guided laser mainly includes heat-force coupling and a chemical reaction synergistic effect: when the high energy density laser beam is focused on the surface of the material through the water jet, the aluminum matrix takes the lead in melting and vaporization due to its low melting point, while the SiC particles undergo oxidation and thermal decomposition at local high temperatures due to high thermal stability. At the same time, the impact force of the water jet will quickly wash the molten aluminum and decomposition products and inhibit thermal diffusion through the cooling effect of water to reduce the oxidation and thermal damage of the matrix. Finally, high precision and low defect microgroove forming are realized.

## 4. Conclusions

In this study, the depth, width, and macroscopic morphology of microgroove machined by a water-jet guided laser under different process parameters was investigated. Additionally, the microstructural evolution and ablation mechanisms of SiC_p_/Al composites were examined. The conclusions are as follows:

(1) During the water-jet guided laser machining of the microgroove of SiCp/Al composites,. With the increase in laser power, the depth of the microgroove increases from 154 μm to 492 μm, the width from 63 μm to 74 μm, and the depth-to-width ratio from 2.45 to 6.62; with the increase in scanning speed, the depth of the microgroove decreases from 525.33 μm to 227.16 μm, and the width from 67.61 μm to 71.02 μm, and the depth-to-width ratio from 7.77 to 3.20. With the increase in water jet pressure, the depth increases from 312.29 μm to 3.20. With the increase in water jet pressure, the depth increased from 312.29 μm to 362.39 μm, the width decreased from 71.59 μm to 62.78 μm, and the depth-to-width ratio increased from 4.38 to 5.77.
(2) Under laser irradiation, Al matrix and SiC particles have different chemical reactions at different temperatures. After the action of the water-jet guided laser, the content of Al in the sediments decreased, while the content of C, Si, and O increased.
(3) Water-jet guided laser processing SiCp/Al composites mainly produces thermal–mechanical coupling and a chemical reaction synergistic effect: the material is melted and vaporized under the action of high energy laser beam, and the SiC particles are oxidized and thermally decomposed at local high temperatures due to high thermal stability; at the same time, under the action of a high pressure water beam, the molten residue and other substances are removed, ensuring the high cleanliness and precision of microgroove processing.

## Figures and Tables

**Table 1:** Physical characteristics parameters of the SiC_p_/Al composites.

Properties	Parameters
Density [kg·m^−3^]	2.88
Elastic modulus [GPa]	95
Tensile strength [MPa]	480
Thermal conductivity [W·m^−1^·C^−1^]	225.3
Specific heat capacity [J·Kg^−1^·°C^−1^]	522.1
particle volume fraction [%]	15

**Table 2:** The processing parameters applied for water-jet guided laser scanning.

Parameter	Value
Wavelength *λ* [nm]	532
Nozzle diameter *d* [µm]	50
Beam height *h* [µm]	30
Repetition frequency *f* [kHz]	10

**Table 3:** The laser processing parameters used in this experiment.

Laser Processing Parameters	Values
Laser power *P* [W]	10, 15, 20, 25
Scanning speed *v* [mm/s]	0.5, 1, 1.5, 2
Water jet pressure *F* [MPa]	15, 20, 25, 30

## Data Availability

The original contributions presented in this study are included in the article material. Further inquiries can be directed to the corresponding author(s).

## References

[1] Ogawa F., Masuda C. (2021). Fabrication and the mechanical and physical properties of nanocarbon-reinforced light metal matrix composites: A review and future directions. Mater. Sci. Eng. A-Struct. Mater. Prop. Microstruct. Process..

[2] Du Y., Lu M., Lin J., Li Y., Sun S. (2024). Investigation on machinability of SiCp/Al composites under the synergistic effect of pulsed laser assisted and ultrasonic elliptical vibration cutting. J. Mater. Process. Technol..

[3] Senthil S., Raguraman M., Manalan D.T. (2021). Manufacturing processes & recent applications of aluminium metal matrix composite materials: A review. Mater. Today Proc..

[4] Tzamtzis S., Barekar N.S., Babu N.H., Patel J., Dhindaw B.K., Fan Z. (2009). Processing of advanced Al/SiC particulate metal matrix composites under intensive shearing—A novel rheo-process. Compos. Part A.

[5] Liao Z., Abdelhafeez A., Li H., Yang Y., Diaz O.G., Axinte D. (2019). State-of-the-art of surface integrity in machining of metal matrix composites. Int. J. Mach. Tools Manuf..

[6] Manna A., Bhattacharayya B. (2003). A study on machinability of Al/SiC-MMC. J. Mater. Process. Technol..

[7] Zhang H.-P., Wu Y.-L., Jin J., Liu J., Qu L.-Q. (2023). Optimization of milling tool parameters and experimental study of cryogenic minimum quantity lubrication (CMQL) for SiCp/Al composites. Ferroelectrics.

[8] Wang X., Popov V.L., Yu Z., Li Y., Xu J., Li Q., Yu H. (2022). Preparation of micro-pit-textured PCD tools and micro-turning experiment on SiCp/Al composites. Micromachines.

[9] Zhong Z.W., Hung N.P. (2002). Grinding of alumina/aluminum composites. J. Mater. Process. Technol..

[10] Gu P., Zhu C., Sun Y., Wang Z., Tao Z., Shi Z. (2023). Surface roughness prediction of SiCp/Al composites in ultrasonic vibration-assisted grinding. J. Manuf. Process..

[11] Yingfei G., Jiuhua X., Hui Y. (2010). Diamond tools wear and their applicability when ultra-precision turning of SiCp/2009Al matrix composite. Wear.

[12] Hu T., Yuan S., Wei J., Zhou N., Zhang Z., Zhang J., Li X. (2024). Water jet guided laser grooving of SiCf/SiC ceramic matrix composites. Opt. Laser Technol..

[13] Wei J., Yuan S., Yang S., Gao M., Fu Y., Hu T., Li X., Fan X., Zhang W. (2024). Waterjet-guided laser processing of SiC/SiC ceramic matrix composites to obtain high cleanliness and low oxidation damage characteristics surfaces. Surf. Coat. Technol..

[14] Cheng B., Ding Y., Li Y., Li J.Y., Xu J.J., Li Q., Yang L.J. (2021). Coaxial helical gas assisted laser water jet machining of SiC/SiC ceramic matrix composites. J. Mater. Process. Technol..

[15] Wu Q., Xu W., Zhang L. (2019). Machining of particulate-reinforced metal matrix composites: An investigation into the chip formation and subsurface damage. J. Mater. Process. Technol..

[16] Wu X., Sun D. (2024). Research on SiC/Al laser-assisted nano-cutting based on molecular dynamics simulation. Mater. Today Commun..

